# Supplementary material for: Understanding Electrical Conduction and Nanopore Formation During Controlled Breakdown
Source: Small. 2021 Aug 1;17(37):2102543. doi: 10.1002/smll.202102543 (PMC11475566; doi:10.1002/smll.202102543)
Supplement: Supplementary file 1 — Supporting Information [file SMLL-17-2102543-s001.pdf]

© 2021 Wiley-VCH GmbH

NANO MICRO small

Supporting Information

for *Small,* DOI: 10.1002/smll.202102543

Understanding Electrical Conduction and Nanopore Formation During Controlled Breakdown

Jasper P. Fried, Jacob L. Swett, Binoy Paulose Nadappuram, Aleksandra Fedosyuk, Pedro Miguel Sousa, Dayrl P. Briggs, Aleksandar P. Ivanov, Joshua B. Edel, Jan A. Mol, and James R. Yates*

Supporting Information:

Understanding Electrical Conduction and Nanopore Formation During Controlled Breakdown

Jasper P. Fried[1], Jacob L. Swett[1], Binoy Paulose Nadappuram[2], Aleksandra Fedosyuk[2], Pedro Miguel Sousa[3], Dayrl P. Briggs[4], Aleksandar P. Ivanov[2], Joshua B. Edel[2], Jan A. Mol[5], James R. Yates[3]

[1] *Department of Materials, University of Oxford, Oxford OX1 3PH, U.K.*

[2] *Department of Chemistry, Imperial College London, Molecular Science Research Hub, White City Campus, 82 Wood Lane, W12 0BZ, U.K.*

[3] *Instituto de Tecnologia Química e Biológica António Xavier, Universidade Nova de Lisboa, Av. da República, 2780-157 Oeiras, Portugal*

[4] *Center for Nanophase Materials Science, Oak Ridge National Laboratory, Oak Ridge, TN 37830, U.S.A*

[5] *Department of Physics, Queen Mary University, London, E1 4NS, U.K.*

Email: jasper.fried@materials.ox.ac.uk, j.yates@itqb.unl.pt, j.mol@qmul.ac.uk

Contents

1 Description of Wafers Used 3

2 Film Deposition Parameters 4

3 Temperature Dependence of Conduction in MIM Devices 5

4 Space Charge Effects in MIM Devices 6

5 More Examples of Conduction in Forward and Reverse Biased MIE Devices 7

6 Breakdown Voltage of MIM and MIE Forward Biased Devices 8

7 More Examples of CBD with Microelectrodes on the Membrane Surface 9

8 More Examples of Conduction in EIE Devices When Varying the Membrane Stoichiometry 10

9 MIE devices for stoichiometric SiN_x membranes 11

10 Plots of conduction and breakdown on a different scales 12

1 Description of Wafers Used

Table 1 shows a description of wafers used in this paper as well as what experiment each wafer was used for.

**Wafer No.**	**Material Stack**	**Suspended Area**	**N:Si ratio**	**Experiments Used In**	**Additional Notes**
Wafer 1	Si (300 μm)<br>SiO_2 (500 nm)<br>SiN_x (25 nm)	57x17 μm^2	1.14	Fig. 2<br>Fig. 3<br>Fig. 5(a) (x=1.14)<br>Fig. 7	None
Wafer 2	Si (300 μm)<br>SiO_2 (500 nm)<br>SiN_x (20 nm)	50x88 μm^2	1.14	Fig. 4	Electrodes deposited before etching of the substrate via photolithography and electron beam lithography (as described in the Methods)
Wafer 3	Si (300 μm)<br>SiO_2 (500 nm)<br>SiN_x (21 nm)	42x42 μm^2	1.06	Fig. 5 (x=1.06)	None
Wafer 4	Si (300 μm)<br>SiO_2 (500 nm)<br>SiN_x (23 nm)	47x47 μm^2	1.33	Fig. 5 (x=1.33)	None

Table 1: Properties of the different wafers used in this study as well as what experiments each wafer was used for.

2 Film Deposition Parameters

Table 2 shows a description of the deposition parameters used for the wafers shown in this paper.

**Wafer No.**	**Time (min:sec)**	**Temp** (°C)	**SiH_2Cl_2 Flow Rate (sccm)**	**NH_3 Flow Rate (sccm)**	**Pressure (MTorr)**
Wafer 1	03:45	852	100	25	220
Wafer 2	03:00	852	100	25	220
Wafer 3	04:08	852	100	20	220
Wafer 4	08:20	775	26	78	200

Table 2: Deposition parameters for the different wafers used in this study.

3 Temperature Dependence of Conduction in MIM Devices

Figure 1 shows the measured current through a SiN_x membrane in a MIM device for three different membrane temperatures. To control the membrane temperature the device was wirebonded to a printed circuit board which was placed on a hot plate and approximately half an hour allowed to pass before performing measurements (to allow the system to reach equilibrium). As can be seen in Fig. 1(a) there is a clear increase in the conduction through the membrane with increasing temperature. In Fig. 1(b) we plot the natural log of the current density against the inverse temperature as calculated at a voltage of 9.6 V. The data shows a linear relationship as expected from Eq. 1 in the main manuscript. Note that each measurement was performed on a device that has not previously been exposed to electric fields to avoid uncertainties related to charge accumulation as discussed in the body of the manuscript.

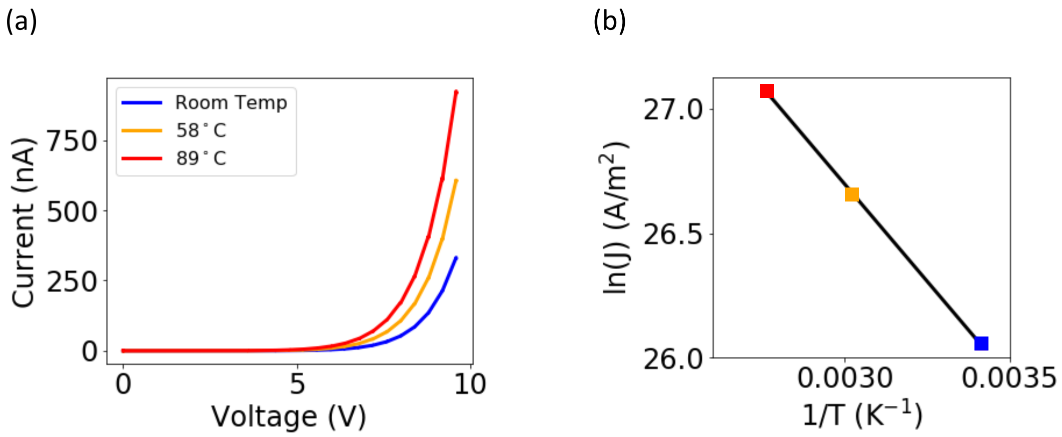

Figure 1: (a) Plot of the conduction through MIM devices for three different membrane temperatures. (b) Plot of the natural log of the current density against the inverse temperature. The plot is linear as expected from Eq. 1 in the manuscript (black line is a linear fit to the data).

4 Space Charge Effects in MIM Devices

Figure 2(a) shows the measured current after the applied voltage is changed from 0 V to 10 V. An exponential fit to this data is shown by the grey dashed line. The time constant for the exponential decay based on the fit is $\sim$55.1 s. By applying a triangular wave voltage and measuring the resulting current we can extract the capacitance of the device (i.e. $C = I(dV/dt)^{-1}$). This is done in Fig. 2(b) where we have applied a triangular wave voltage with frequency $f = 500$ Hz and amplitude $V_{PP} = 50$ mV and extracted a device capacitance of 70 pF based on the resulting current. Assuming a resistance of the leads of 100 Ω, the expected RC time constant is 70 ns. This is much smaller than that observed in our experiments, suggesting significant space charge effects are present.

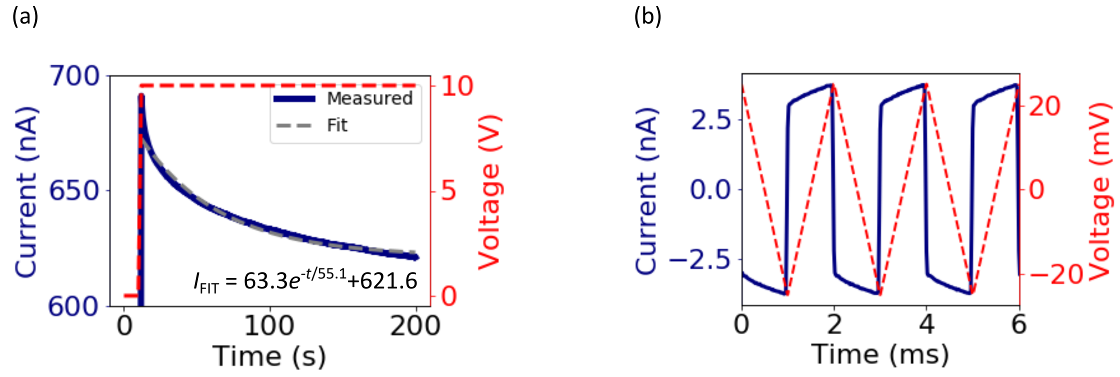

Figure 2: (a) Slow changes in the measured current in response to changing the voltage from 0 V to 10 V. An exponential fit to the data is shown by the grey dashed line. (b) Resulting current when applying a triangular wave voltage ($f = 500$ Hz, $V_{pp} = 50$ mV) across the membrane. Based on the measured current the capacitance can be estimated at 70 pF.

5 More Examples of Conduction in Forward and Reverse Biased MIE Devices

Figure 3 shows more examples of Poole-Frenkel (PF) plots of the conduction through MIE in the forward and reverse biased configurations. The results reported in the body of the paper are consistent across multiple devices.

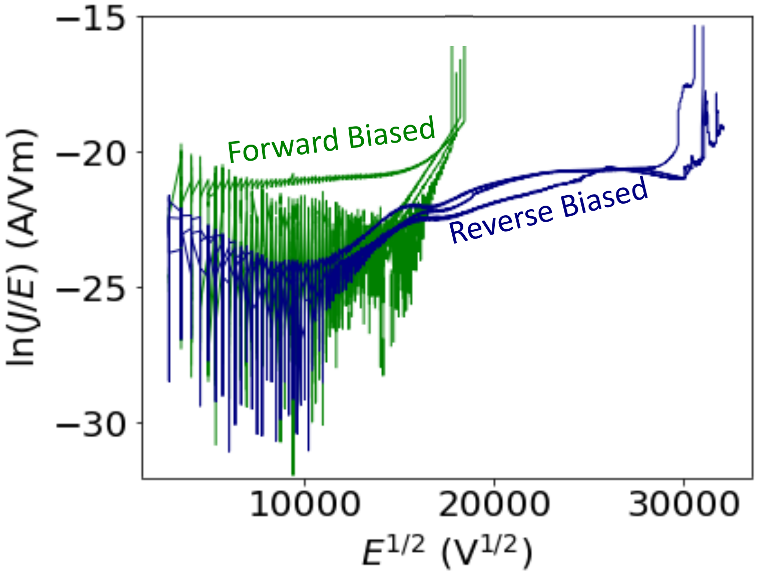

Figure 3: PF plot showing conduction and breakdown in several MIE devices in the foward and reverse biased configurations.

6 Breakdown Voltage of MIM and MIE Forward Biased Devices

Figure 4(a) shows a PF plot comparing conduction and breakdown in several MIM and MIE devices in the forward biased configuration. Figure 4(b) shows the same data, however, here we have plotted the measured current as a function of the applied voltage. While the conduction behaviour before breakdown is similar for both device geometries, the MIE devices consistently breakdown at a lower voltage (and therefore current density). This result is consistent across all measured devices.

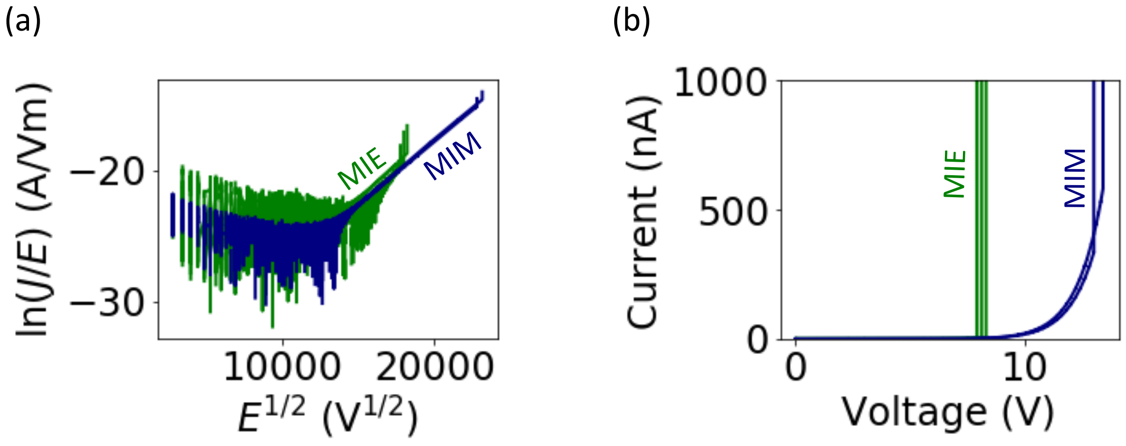

Figure 4: (a) PF plot comparing conduction and breakdown in MIM and MIE devices in the forward biased configuration. (b) The same data as (a), however, here we have plotted the measured current against the applied voltage.

7 More Examples of CBD with Microelectrodes on the Membrane Surface

Figure 5 shows more examples of fluorescent imaging of the position of nanopores created when performing CBD on devices with microelectrodes on the membrane surface. For each configuration a time series of data is shown with a frame before, during, and after the application of a voltage which drives Ca^{2+} ions through the nanopore. Consistent with the discussion in the body of the paper, for the forward biased configuration, nanopores form only within the area covered by the electrodes on the membrane surface. For the reverse biased configuration, nanopores form at random locations in the membrane.

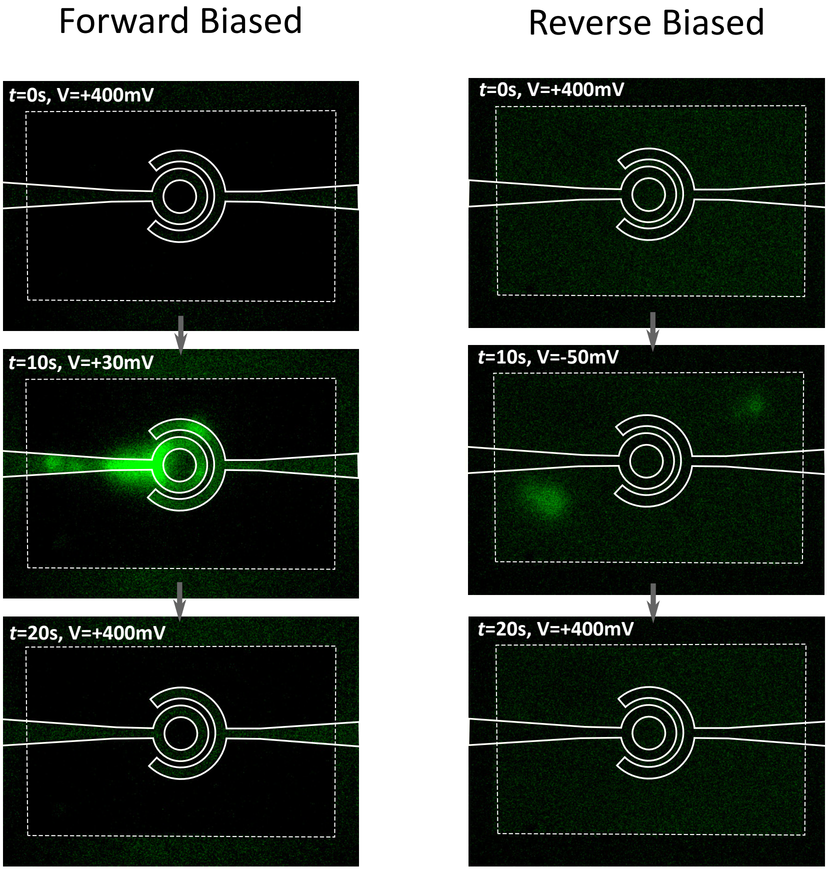

Figure 5: Further examples of fluorescent micrographs showing the position of nanopores created when CBD is performed on devices with microelectrodes on the membrane surface.

8 More Examples of Conduction in EIE Devices When Varying the Membrane Stoichiometry

Figure 6 shows more examples of conduction and breakdown in EIE devices for a Si-rich SiN_x (x=1.06) and a stoichiometric SiN_x (x=1.33). Conduction characteristics are dependent upon membrane stoichiometry and are reproducible for each batch of devices.

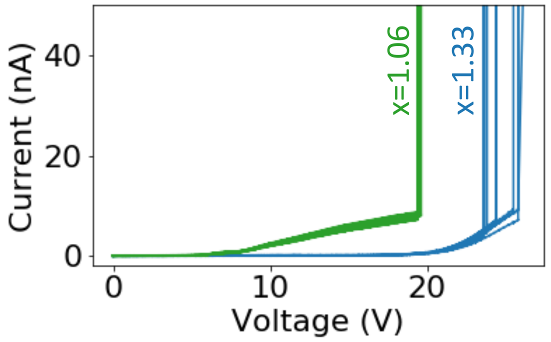

Figure 6: Examples of conduction and breakdown in EIE for a Si-rich SiN_x (x=1.06) and a stoichiometric SiN_x (x=1.33).

9 MIE devices for stoichiometric SiN_x membranes

Figure 7 shows a comparison of conduction and breakdown in forward and reverse-biased MIE devices for a stoichiometric SiN_x membrane. The conduction characteristics are observed to be relatively similar. This is consistent with the fact that oxidation reactions at the membrane electrolyte interface have a reduced effect on the conduction for these membranes. Rather, conduction is predominately limited by charge transport across the dielectric for the stoichiometric Si_3N_4. Despite the similar conduction characteristics, it is observed that breakdown occurs at a lower voltage (and therefore current) for the MIE device in the forward-biased configuration. This is similar to the result discussed in the body of the manuscript that showed MIE devices in the forward biased configuration breakdown at a lower voltage than MIM devices. This may be associated with the presence of H^+ ions at the membrane-electrolyte interface, however, the mechanism for this is not currently understood.

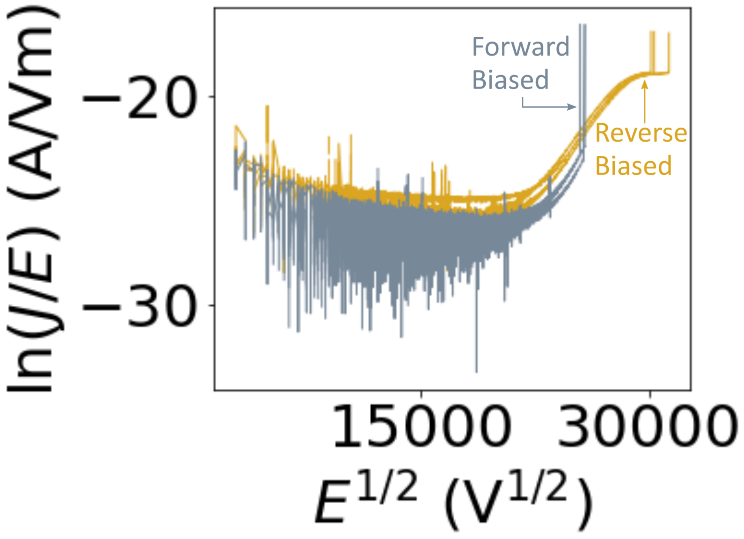

Figure 7: Comparison of conduction and breakdown in forward and reverse-biased devices for a stoichiometric SiN_x membrane.

10 Plots of conduction and breakdown on a different scales

Figure 8 shows plots of several of the figures shown in the main manuscript using a logarithmic scale for the measured current. This may be useful to observe the conduction characteristics in some cases.

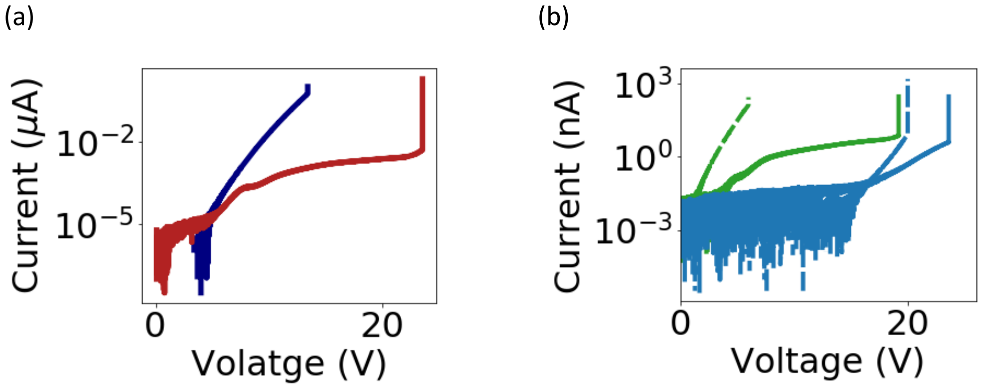

Figure 8: Plots of several of the figures shown in the main manuscript using a logarithmic scale for the current. (a) Shows the same data as Fig 2(c) in the main manuscript with (b) shows the same data as Fig 5(b) in the manuscript.

Figure 9 shows plots of several of the figures shown in the main manuscript with a zoomed y-scale to highlight the current prior to breakdown.

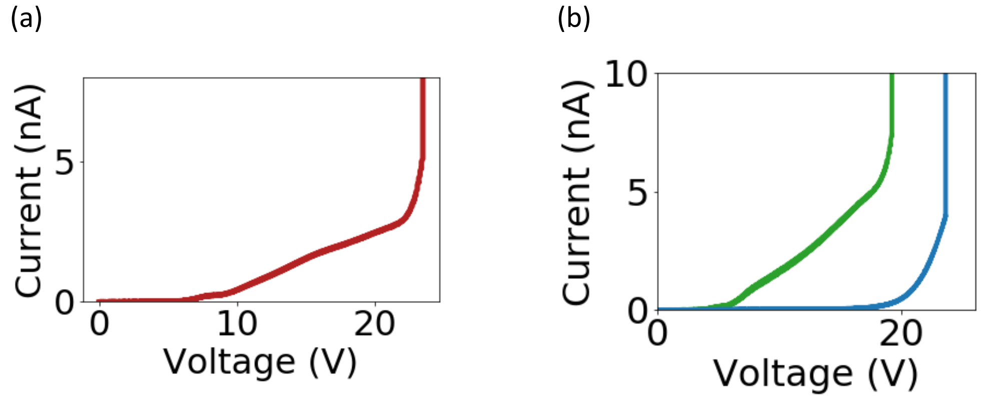

Figure 9: Plots of several of the figures shown in the main manuscript with a reduced y-scale to show the conduction prior to breakdown. (a) Shows the same data as Fig 2(c) in the main manuscript with (b) shows the same data as Fig 5(b) in the manuscript. Only the conduction in EIE devices are shown for the zooms.
